# Recurrent Status Epilepticus in an Adult With Spastic Quadriplegic Cerebral Palsy

**DOI:** 10.7759/cureus.101335

**Published:** 2026-01-12

**Authors:** Ashraf Mukhtar, Mutasim Binidris, Moayad H Ali, Mohammed Kuttub Udin, Moayad A Elgassim

**Affiliations:** 1 Department of Medical Education, Hamad Medical Corporation, Doha, QAT; 2 School of Medicine, Taylor's University, Selangor, MYS; 3 Department of Emergency Medicine, Hamad Medical Corporation, Doha, QAT

**Keywords:** cerebral palsy, epilepsy, phenytoin, status epilepticus, valproate

## Abstract

Status epilepticus (SE) is a neurological emergency associated with high morbidity and mortality. Management of individuals with cerebral palsy (CP) presents unique challenges due to baseline neurological deficits, limited communication, and altered metabolism of antiepileptic drugs (AEDs). We report the case of a 22-year-old male with spastic quadriplegic CP and long-standing epilepsy who presented with recurrent generalized tonic-clonic seizures persisting for five hours despite medication compliance. His pre-existing neurological impairment complicated early recognition. A non-contrast CT brain ruled out intracranial hemorrhage, stroke, and infection. The patient was treated with intravenous diazepam and phenytoin, resulting in seizure cessation within two hours, and was discharged seizure-free after four days. This report highlights the importance of prompt diagnosis and timely escalation of treatment in SE in adults with CP. Multidisciplinary follow-up and caregiver education remain essential for long-term control and prevention of recurrence.

## Introduction

Status epilepticus (SE) is defined by the International League Against Epilepsy (ILAE) as either a seizure lasting five minutes or longer, or the occurrence of two or more discrete seizures without recovery of consciousness between episodes [1]. It is a neurological emergency associated with significant morbidity and mortality if treatment is delayed [2]. Cerebral palsy (CP) refers to a group of permanent disorders of movement and posture that cause activity limitation, attributed to non-progressive disturbances occurring in the developing fetal or infant brain [3]. Epilepsy is reported in up to 50% of individuals with CP, with approximately 10% developing SE at least once during their lifetime [4]. Adults with CP and epilepsy face unique management challenges due to abnormal cortical architecture, reduced drug absorption, and altered hepatic metabolism of antiepileptic drugs (AEDs) [5].

Advances in neonatal and supportive care have led to improved survival of individuals with CP into adulthood, thereby shifting the burden of care toward adult neurological complications. In this population, recognition of SE may be delayed due to baseline motor impairment, communication limitations, and atypical presentations of seizures. We report a case of recurrent SE in an adult with spastic quadriplegic CP, unremarkable neuroimaging, and recurrence attributed to sub-therapeutic antiepileptic drug (AED) levels.

## Case presentation

A 22-year-old man with spastic quadriplegic CP and epilepsy since early childhood presented to the emergency department with recurrent generalized tonic-clonic seizures lasting approximately five hours. Each seizure involved bilateral limb stiffening followed by clonic movements lasting one to two minutes with progressively shorter inter-ictal intervals. There had been no preceding fever, trauma, vomiting, or infection. Based on continuous seizure activity with progressively shortening inter-ictal periods lasting approximately five hours without return to baseline consciousness, the patient met the clinical criteria for convulsive SE. He was compliant with syrup valproate 400 mg twice daily and baclofen 10 mg three times daily. His last hospital admission for SE had occurred one year prior, following sleep deprivation. He was fully dependent for activities of daily living, lived with his parents, and had no family history of epilepsy.

**Figure 1:**
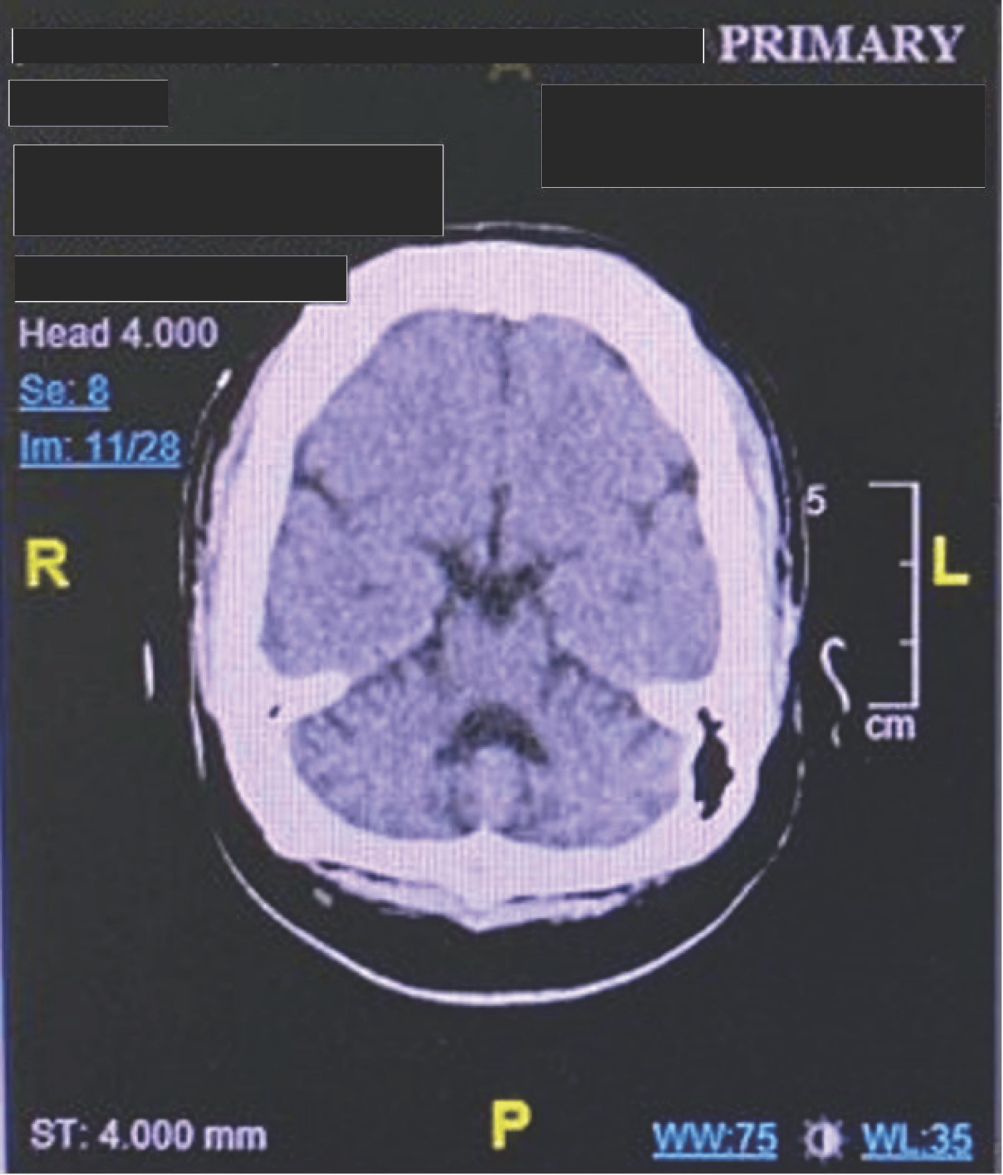
Non-contrast CT brain demonstrating preserved gray-white matter differentiation Contrast-enhanced imaging was not performed; therefore, leptomeningeal enhancement was not directly assessed CT: computed tomography

Upon arrival, the patient was postictal but arousable. According to family members, this level of reduced responsiveness was atypical compared with his usual postictal recovery. Vital signs were as follows: temperature: 37 °C, pulse: 72 beats per minute, blood pressure: 130/79 mmHg, respiratory rate: 18 breaths per minute, and oxygen saturation: 97% on room air. Neurological examination revealed hypertonia and hyperreflexia in all limbs with bilateral ankle clonus, consistent with spastic quadriplegia. Cranial nerves were intact, and no meningeal signs were observed. Cardiovascular, respiratory, and abdominal examinations were unremarkable. A non-contrast CT of the brain performed on admission showed no acute intracranial abnormality, with preserved gray-white matter differentiation and no evidence of intracranial hemorrhage, infarction, mass lesion, or midline shift (Figure 1).

These findings excluded hemorrhagic stroke, trauma, or infection as causes of SE [5]. Cardiac and respiratory evaluations were performed to rule out systemic triggers. A 12-lead electrocardiogram (ECG) showed normal sinus rhythm with no ischemic or arrhythmic changes, and a chest radiograph demonstrated clear lung fields with a normal cardiac silhouette, excluding aspiration or pneumonia.

Routine laboratory investigations were largely normal except for a sub-therapeutic valproate level (Table 1). Given the absence of fever, leukocytosis, meningeal signs, or radiologic features suggestive of intracranial infection, an infectious etiology was considered unlikely.

**Table 1:** Admission laboratory findings and interpretation ^*^Abnormal value AST: aspartate transaminase; ALT: alanine transaminase; ALP: alkaline phosphatase

Test	Result	Reference range
Hemoglobin	13.7 g/dL	13–17 g/dL
White blood cell count	7.8 × 10⁹/L	4–11 × 10⁹/L
Platelet count	275 × 10⁹/L	150–400 × 10⁹/L
Sodium	139 mmol/L	135–145 mmol/L
Potassium	4.1 mmol/L	3.5–5.0 mmol/L
Chloride	103 mmol/L	98–107 mmol/L
Bicarbonate	24 mmol/L	22–29 mmol/L
Urea	5.1 mmol/L	2.5–7.5 mmol/L
Creatinine	78 µmol/L	60–110 µmol/L
AST	28 U/L	<40 U/L
ALT	24 U/L	<41 U/L
ALP	87 U/L	45–129 U/L
Total bilirubin	9 µmol/L	<21 µmol/L
Random glucose	5.3 mmol/L	3.9–7.8 mmol/L
Serum valproate	45 µg/mL^*^	50–100 µg/mL

After stabilization, an electroencephalogram (EEG) was performed to evaluate cerebral activity. The tracing revealed diffuse slowing and disorganized background rhythms consistent with postictal encephalopathy (Table 2), without focal spikes or epileptiform discharges. As the EEG was performed after clinical stabilization and continuous EEG monitoring was unavailable, these findings do not exclude preceding nonconvulsive SE.

**Table 2:** EEG report obtained after stabilization The report shows diffuse slowing and disorganized background activity consistent with postictal encephalopathy EEG: electroencephalogram; Cps: cycles per second

Waves	Frequency (cps)	% of time	EEG pattern/interpretation
Alpha waves	8–10, normal (8–13)	10–15%	Occasional posterior dominant rhythm, poorly sustained
Beta waves	15–20, normal (14–30)	20–25%	Diffuse, symmetrical, low amplitude
Theta waves	4–6, normal (4–8)	40–50%	Diffuse, symmetrical, increased prominence over temporal regions
Delta waves	1–3, normal (0.5–4)	25–30%	Diffuse slowing, low voltage, disorganized background

The patient was managed with intravenous diazepam 10 mg, repeated once after five minutes, followed by intravenous phenytoin 15 mg/kg (1 g total) over 20 minutes, then maintenance phenytoin 100 mg every eight hours. Oral valproate dose was increased to 600 mg twice daily. Following the initiation of treatment, seizure frequency progressively decreased, with complete cessation within approximately two hours of presentation. As seizures responded to first- and second-line therapy, further escalation was not required.

The patient was discharged on combined valproate-phenytoin therapy with neurology follow-up. Two weeks later, he remained seizure-free and alert. Follow-up laboratory testing confirmed therapeutic drug levels and metabolic stability (Table 3).

**Table 3:** Follow-up laboratory results showing therapeutic antiepileptic levels and normal metabolic profile All follow-up parameters remained within normal limits, confirming adequate drug control and organ function AST: aspartate transaminase; ALT: alanine transaminase

Parameter	Result	Reference range
Serum valproate	82 µg/mL	50–100 µg/mL
Serum phenytoin	14 µg/mL	10–20 µg/mL
Sodium	140 mmol/L	135–145 mmol/L
Potassium	4.2 mmol/L	3.5–5.0 mmol/L
Urea	4.8 mmol/L	2.5–7.5 mmol/L
Creatinine	76 µmol/L	60–110 µmol/L
ALT	26 U/L	<41 U/L
AST	30 U/L	<40 U/L
Calcium	2.35 mmol/L	2.10–2.55 mmol/L

## Discussion

This report highlights the diagnostic and therapeutic complexity of SE in adults with spastic quadriplegic CP. Despite normal structural neuroimaging, significant functional derangements in neuronal excitability can trigger prolonged seizure activity. A normal CT brain, therefore, does not exclude severe electrophysiological dysfunction, and clinicians must remain alert to functional or metabolic causes [6-8]. The ILAE strongly recommends urgent neuroimaging in SE to exclude secondary pathology, yet normal imaging is common in patients with underlying epilepsy or developmental disorders [9]. In this case, the absence of focal lesions redirected attention toward AED serum levels and systemic contributors, which proved sub-therapeutic. Such interpretation of “negative” imaging prevents diagnostic inertia and ensures prompt pharmacologic correction.

EEG remains the most valuable diagnostic adjunct once the patient is stabilized. Importantly, because the EEG in this case was obtained after seizure control, the absence of epileptiform discharges should not be interpreted as exclusion of prior nonconvulsive status epilepticus, highlighting the diagnostic limitations of post-treatment EEG in delayed presentations. Our patient’s low-voltage background with predominant theta-delta activity was characteristic of postictal encephalopathy rather than persistent ictal discharge [10]. Recognizing these transient electrical changes can help avoid unnecessary escalation of therapy and confirm neurological recovery.

Pathophysiologically, SE results from failure of inhibitory GABAergic transmission alongside sustained glutamatergic excitation, producing neuronal injury and systemic stress [11]. Timely benzodiazepine administration remains the cornerstone of initial management, followed by second-line long-acting AEDs such as phenytoin, valproate, or levetiracetam to prevent recurrence [12-14]. The patient’s excellent response to combined diazepam and phenytoin is consistent with evidence-based algorithms for convulsive SE [14]. Adults with CP represent a special pharmacologic subset. Altered drug absorption from spastic gastrointestinal musculature, reduced hepatic clearance, and interactions with agents such as baclofen complicate maintenance therapy [15,16]. Baclofen’s potential to lower seizure threshold shows the importance of balancing antispastic and antiepileptic regimens. Furthermore, irregular oral intake and polypharmacy can lead to unpredictable serum concentrations, emphasizing the need for periodic therapeutic drug monitoring.

Beyond pharmacology, prevention is a multidisciplinary challenge. Consistent medication adherence, caregiver training for early use of rescue benzodiazepines, and lifestyle modifications such as adequate sleep, hydration, and avoidance of known triggers significantly reduce recurrence [17-19]. Incorporating community-based epilepsy care programs and caregiver education can further minimize emergency admissions and improve long-term neurological outcomes. Finally, this report reinforces that SE management in adults with CP should extend beyond acute stabilization. Regular multidisciplinary follow-up integrating neurology, rehabilitation medicine, nutrition, and social support optimizes seizure control and preserves quality of life [20].

## Conclusions

This report demonstrates that adults with CP remain at measurable risk for new or breakthrough seizures and even SE, despite long-term stability and the absence of acute neuroimaging abnormalities. The normal CT findings in this patient show that seizure recurrence in this population is not always structural in origin but may stem from altered cortical excitability, medication non-adherence, metabolic fluctuations, or lowered seizure thresholds associated with comorbidities and chronic neuromotor impairment. Clinically, this reinforces the need for physicians to maintain high suspicion for subtle or evolving seizure activity in patients with CP, to promptly perform EEG monitoring when indicated, and to individualize AED regimens rather than relying solely on radiologic reassurance. The favorable neurological recovery observed following timely escalation of therapy demonstrates the effectiveness of rapid multidisciplinary management and medication optimization. Overall, this report adds to the increasing awareness that proactive, patient-specific surveillance and early therapeutic adjustments are crucial to preventing neurological decline and improving long-term seizure outcomes in adults with chronic neurodevelopmental disorders.

## References

[1] Trinka E, Cock H, Hesdorffer D (2015). A definition and classification of status epilepticus—report of the ILAE Task Force on classification of status epilepticus. Epilepsia.

[2] Ascoli M, Ferlazzo E, Gasparini S, Mastroianni G, Citraro R, Roberti R, Russo E (2021). Epidemiology and outcomes of status epilepticus. Int J Gen Med.

[3] Rosenbaum P, Paneth N, Leviton A (2007). A report: the definition and classification of cerebral palsy April 2006. Dev Med Child Neurol Suppl.

[4] Sellier E, Uldall P, Calado E, Sigurdardottir S, Torrioli MG, Platt MJ, Cans C (2012). Epilepsy and cerebral palsy: characteristics and trends in children born in 1976-1998. Eur J Paediatr Neurol.

[5] Pérez IF, Villagra TB, Jiménez-Balado J, Redondo JJ, Recasens BB (2023). Risk factors and outcome of epilepsy in adults with cerebral palsy or intellectual disability. Epilepsy Behav.

[6] Bosque Varela P, Machegger L, Crespo Pimentel B, Kuchukhidze G (2025). Imaging of status epilepticus. J Clin Med.

[7] Nair PP, Kalita J, Misra UK (2009). Role of cranial imaging in epileptic status. Eur J Radiol.

[8] Cascino GD (1993). Nonconvulsive status epilepticus in adults and children. Epilepsia.

[9] Wylie T, Sandhu DS, Murr NI (2025). Status Epilepticus. StatPearls.

[10] Kaplan PW (2007). EEG criteria for nonconvulsive status epilepticus. Epilepsia.

[11] Wasterlain CG, Fujikawa DG, Penix L, Sankar R (1993). Pathophysiological mechanisms of brain damage from status epilepticus. Epilepsia.

[12] Glauser T, Shinnar S, Gloss D (2016). Evidence-based guideline: treatment of convulsive status epilepticus in children and adults: report of the Guideline Committee of the American Epilepsy Society. Epilepsy Curr.

[13] Brophy GM, Bell R, Claassen J (2012). Guidelines for the evaluation and management of status epilepticus. Neurocrit Care.

[14] Hirsch LJ, Claassen J (2002). The current state of treatment of status epilepticus. Curr Neurol Neurosci Rep.

[15] Sesse A, Ladias P, Kostoulas C, Chatzistefanidis D, Georgiou I, Markoula S (2025). Metabolic pathways and genes involved in treatable and non-treatable metabolic epilepsies. A comprehensive review and metabolic pathway analysis. Metab Brain Dis.

[16] Hyder Pottoo F, Salahuddin M, Khan FA (2022). Trio-drug combination of sodium valproate, baclofen and thymoquinone exhibits synergistic anticonvulsant effects in rats and neuroprotective effects in HEK-293 cells. Curr Issues Mol Biol.

[17] Sculier C, Gaínza-Lein M, Sánchez Fernández I, Loddenkemper T (2018). Long-term outcomes of status epilepticus: a critical assessment. Epilepsia.

[18] Akinbi MS, Welty TE (1999). Benzodiazepines in the home treatment of acute seizures. Ann Pharmacother.

[19] Löscher W, Potschka H, Sisodiya SM, Vezzani A (2020). Drug resistance in epilepsy: clinical impact, potential mechanisms, and new innovative treatment options. Pharmacol Rev.

[20] Novak I, Morgan C, Fahey M (2020). State of the evidence traffic lights 2019: systematic review of interventions for preventing and treating children with cerebral palsy. Curr Neurol Neurosci Rep.

